# Progeny Varicella-Zoster Virus Capsids Exit the Nucleus but Never Undergo Secondary Envelopment during Autophagic Flux Inhibition by Bafilomycin A1

**DOI:** 10.1128/JVI.00505-19

**Published:** 2019-08-13

**Authors:** James H. Girsch, Katherine Walters, Wallen Jackson, Charles Grose

**Affiliations:** a Department of Microbiology and Immunology, University of Iowa, Iowa City, Iowa, USA; b Central Microscopy Research Core, University of Iowa, Iowa City, Iowa, USA; c Division of Infectious Diseases/Virology, Children’s Hospital, University of Iowa, Iowa City, Iowa, USA; University of California, Irvine

**Keywords:** LC3-II, autophagy, herpes simplex virus, multivesicular body, pseudorabies, *trans*-Golgi network

## Abstract

Varicella-zoster virus (VZV) is an alphaherpesvirus that lacks the herpesviral neurovirulence protein ICP34.5. The underlying hypothesis of this project was that inhibitors of autophagy reduce VZV infectivity. We selected the vacuolar proton ATPase inhibitor bafilomycin A1 for analysis because of its well-known antiautophagy property of impeding acidification during the late stage of autophagic flux. We documented that bafilomycin treatment from 48 to 72 h postinfection lowered VZV titers substantially (*P* ≤ 0.008). Because we were unable to define the site of the block in the infectious cycle by confocal microscopy, we turned to electron microscopy. Capsids were observed in the nucleus, in the perinuclear space, and in the cytoplasm adjacent to Golgi apparatus vesicles. Many of the capsids had an aberrant appearance, as has been observed previously in infections not treated with bafilomycin. In contrast to prior untreated infections, however, secondary envelopment of capsids was not seen in the *trans*-Golgi network, nor were prototypical enveloped particles with capsids (virions) seen in cytoplasmic vesicles after bafilomycin treatment. Instead, multiple particles with varying diameters without capsids (light particles) were seen in large virus assembly compartments near the disorganized Golgi apparatus. Bafilomycin treatment also led to increased numbers of multivesicular bodies in the cytoplasm, some of which contained remnants of the Golgi apparatus. In summary, we have defined a previously unrecognized property of bafilomycin whereby it disrupted the site of secondary envelopment of VZV capsids by altering the pH of the *trans*-Golgi network and thereby preventing the correct formation of virus assembly compartments.

**IMPORTANCE** This study of VZV assembly in the presence of bafilomycin A1 emphasizes the importance of the Golgi apparatus/*trans*-Golgi network as a platform in the alphaherpesvirus life cycle. We have previously shown that VZV induces levels of autophagy far above the basal levels of autophagy in human skin, a major site of VZV assembly. The current study documented that bafilomycin treatment led to impaired assembly of VZV capsids after primary envelopment/de-envelopment but before secondary reenvelopment. This VZV study also complemented prior herpes simplex virus 1 and pseudorabies virus studies investigating two other inhibitors of endoplasmic reticulum (ER)/Golgi apparatus function: brefeldin A and monensin. Studies with porcine herpesvirus demonstrated that primary enveloped particles accumulated in the perinuclear space in the presence of brefeldin A, while studies with herpes simplex virus 1 documented an impaired secondary assembly of enveloped viral particles in the presence of monensin.

## Abstract (precis)

This study of VZV assembly in the presence of bafilomycin A1 emphasizes the importance of the Golgi apparatus/*trans*-Golgi network as a platform in the alphaherpesvirus life cycle. We have previously shown that VZV induces levels of autophagy far above the basal levels of autophagy in human skin, a major site of VZV assembly. The current study documented that bafilomycin treatment led to impaired assembly of VZV capsids after primary envelopment/de-envelopment but before secondary reenvelopment. This VZV study also complemented prior herpes simplex virus 1 and pseudorabies virus studies investigating two other inhibitors of endoplasmic reticulum (ER)/Golgi apparatus function: brefeldin A and monensin. Studies with porcine herpesvirus demonstrated that primary enveloped particles accumulated in the perinuclear space in the presence of brefeldin A, while studies with herpes simplex virus 1 documented an impaired secondary assembly of enveloped viral particles in the presence of monensin.

## INTRODUCTION

Macroautophagy (here called autophagy) is the subject of many recent articles by virologists. With regard to herpesviruses, interest was galvanized by the insightful observation that the herpes simplex virus 1 (HSV-1) neurovirulence protein ICP34.5 bound to Beclin 1, thereby inhibiting maturation of the autophagosome (1, 2). Because several closely related herpesviruses, including varicella-zoster virus (VZV), do not contain an ICP34.5 gene homolog in their genomes (3), autophagy studies were pursued with this virus to investigate differences from HSV-1. Based on the HSV-1 data, an early hypothesis stated that an autophagic response would impair VZV replication.

One of the initial methods used to detect autophagy was microscopy (4). The autophagosome is characterized by its double-walled outer membrane containing microtubule-associated protein 1 light chain 3B (LC3-II), the lipidated form of LC3-I (5). Because of the diameter of the autophagosome (300 to 1,000 nm) and its immunogenic LC3-II protein, confocal microscopy with a fluorescent-antibody probe is an excellent method by which to visualize and enumerate autophagosomes in VZV-infected cells (6). Using this technology, we documented an abundant autophagy response in the skin vesicles within the exanthem of human subjects with herpes zoster (7). We have reproduced these results both in infected cell cultures and in infected human skin xenografts within the severe combined immunodeficient (SCID) mouse model for varicella (8, 9). More recently, these results have been duplicated in a human skin organ culture model for herpes zoster infection (10). In another set of experiments, we documented autophagic flux in VZV-infected cells (9). In other words, the accumulation of autophagosomes was not caused by a block in the autophagy pathway. These VZV data are supported by papers that report similar autophagy results after infection with the closely related alphaherpesvirus pseudorabies virus (PRV), as well as duck enteritis herpesvirus (11, 12), both of which also lack the herpesvirus ICP34.5 homolog.

Cells exhibit basal levels of autophagy even during herpesvirus infection (13). However, the levels of autophagy induced by VZV infection in both (i) human skin during the VZV disease called herpes zoster (shingles) and (ii) human skin explants in either the skin organ culture model or the severe combined immunodeficient mouse model of VZV infection are far above basal levels (10). Further, we found that inhibition of autophagy diminishes VZV cell-to-cell spread and infectivity (8). We also found evidence that an intact autophagy pathway is required for VZV exocytosis after secondary envelopment (14). Because of these findings, we postulated that treatment of VZV-infected cultures with the antiautophagic flux drug bafilomycin A1 (BAF) would decrease viral titers. BAF is known to interrupt late stages of flux, which include fusion of the autophagosome with both lysosomes and endosomes (15, 16), as well as fusion of an early endosome with a late endosome (17). Although we did find that treatment with BAF diminished VZV titers, the findings by electron microscopy were both unexpected and insightful. We also correlate the inhibitory BAF-related effects during the VZV infectious cycle with published data about the inhibitory effects of brefeldin A and monensin at two other Golgi apparatus locations during PRV and HSV-1 infectious cycles. Thus, this report not only reaffirms the centrality of the Golgi apparatus/*trans*-Golgi network (TGN) as a platform in the alphaherpesvirus infectious cycle, but also expands the antiherpesviral properties of BAF.

(Portions of this research were carried out as part of honors undergraduate thesis research by J. H. Girsch at the University of Iowa.)

## RESULTS

### Effect of bafilomycin on cultured cells before and after VZV infection.

**FIG 1:**
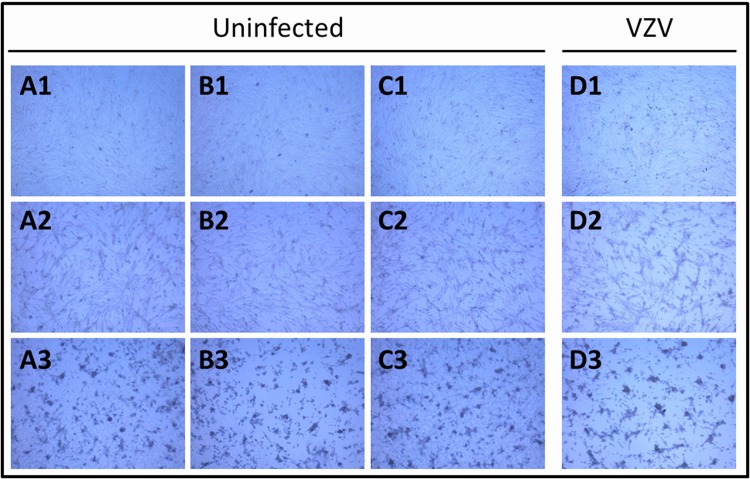
Effects of bafilomycin on uninfected and infected cells. (A to C) Three different monolayers of uninfected MRC5 cells. (D) Infected monolayers. (A1 to D1) No BAF added. (A2 to D2) 10 nm BAF added for 24 h. (A3 to D3) 10 nM BAF added for 48 h. The micrographs were captured with a Nikon Eclipse Ti microscope.

BAF can cause toxicity to cultured cells. Many different concentrations of BAF have been used by virologists in the past 25 years (Table 1). When we reviewed each of the papers listed in Table 1, we found that concentrations equal to or less than 30 nM were commonly selected for experiments longer than a few hours’ duration, whereas higher BAF concentrations for shorter intervals were selected for studies aimed solely at inhibiting viral entry. Because we were not studying VZV entry, we tested the toxic effect of 10 nM BAF on cultured MRC monolayers. BAF was added when the monolayers were approximately 90% confluent. We purposely chose monolayers that were less than confluent because VZV infections are typically carried out in such monolayers (18). The compound was left in the medium for either 24 h or 48 h. Then, the monolayers were visualized by microscopy and photographed. When they were compared with an untreated control monolayer, it was apparent that addition of BAF to the medium inhibited the MRC5 monolayer from reaching confluence (Fig. 1A to C). We also enumerated the cell populations by trypan blue exclusion. Addition of BAF for 24 h decreased the cell count by 35% compared with a control monolayer; after removal of BAF, the cell count remained the same 24 h later. Addition of BAF for 48 h decreased the cell count by 53% compared with a control monolayer; after removal of BAF, the cell count decreased another 4% over the next 24 h.

**TABLE 1:** Bafilomycin A1 concentrations reported in selected virology publications

Virus	BAF concn (nM)	Expt duration (h)	Reference
VZV	10	24, 48	This study
VZV	10	24	49
VZV	100	2.5	48
HSV	50	2.5	58
HSV	100	24	37
Coronavirus	5,000	5	59
Parvovirus	20	16	60
Rhinovirus	20, 200	16	17
Influenza virus	100	1	35
SFV	10, 30	8	34
VSV	100	3	34

Next, we assessed the effects of both VZV infection and BAF treatment on the monolayers (Fig. 1D). BAF was added at either 24 h postinfection (hpi) or 48 hpi; the cultures were photographed at 72 hpi. There was not a notable difference between the appearances of the infected monolayers treated with BAF and the uninfected monolayers treated with BAF; in other words, the cytopathology was primarily due to the BAF treatment rather than the infection. As expected, the cytopathology in both experiments was more extensive after a 48-h incubation with BAF (Fig. 1D). Of note, the degree of BAF-induced cytopathology was more than anticipated after reading the papers listed in Table 1. None of the papers in the table mentioned any observable effects on a monolayer treated with a lower concentration of BAF, such as 10 nM.

### Effects of bafilomycin on LC3-II and VZV gE production.

**FIG 2:**
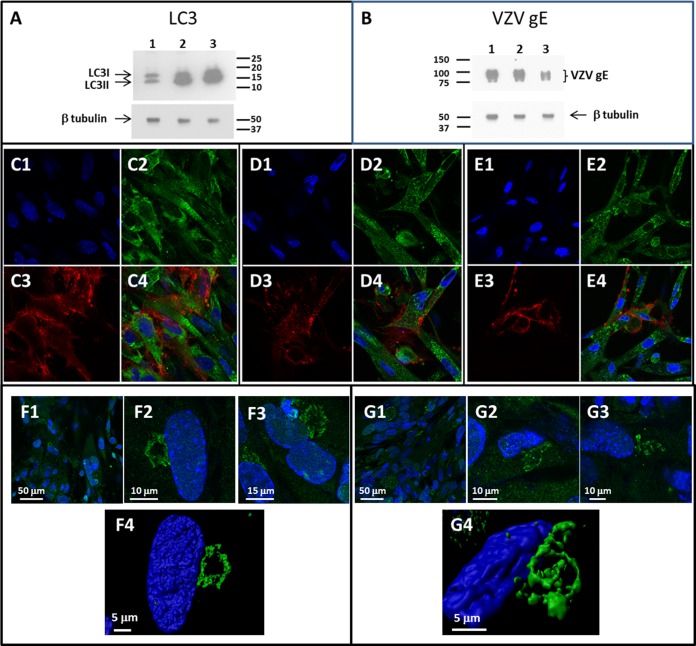
Western blot and confocal analyses of bafilomycin-treated VZV-infected monolayers. (A) Analysis of LC3 in BAF-treated infected monolayers. Infected cells were treated with BAF for 24 h (lane 2) or 48 h (lane 3) or left untreated (lane 1). The cell control was tubulin. (B) Analysis of VZV gE in the virion band after density gradient sedimentation of BAF-treated and untreated monolayers. Infected cells prior to sedimentation were treated with BAF for 24 h (lane 2) or 48 h (lane 3) or left untreated (lane 1). The cell control was tubulin. (C to E) Visualization of infected cells by confocal microscopy in the absence of BAF (C) or in the presence of BAF for 24 h (D) or 48 h (E). Infected cells were labeled with anti-VZV gE MAb (red) (C3, D3, and E3), anti-LC3 antibody (green) (C2, D2, and E2), and Hoechst H33342 dye (blue) (C1, D1, and E1). (C4, D4, and E4) Merge of all labeled channels. (F and G) Visualization of Golgi apparatus by confocal microscopy in infected cells in the absence (F) or presence (G) of BAF. Three 2D images are shown after probing with anti-GM130 MAb (green) (F1 to F3 and G1 to G3). (F4 and G4) One z-stack of images each from panel F and panel G was converted into a 3D image with Imaris software.

Because of the known property of BAF to inhibit autophagic flux, we predicted that the production of LC3-II would be increased in BAF-treated cultures. This prediction was confirmed (Fig. 2A). We also predicted that the biosynthesis of the VZV glycoproteins would be decreased after BAF treatment of infected cultures. To carry out this experiment, we infected 6 75-cm^2^ monolayers with equal numbers of infected cells and then treated 2 of the 6 monolayers with BAF between 48 and 72 hpi and 2 monolayers with BAF between 24 and 72 hpi. Virus was purified by two sequential sedimentations as described in Materials and Methods, after which the three virus bands were analyzed for the predominant viral glycoprotein VZV gE (Fig. 2B). As predicted, compared with the untreated control infected culture, the relative amount of gE declined in monolayers treated with BAF, especially for 48 h. As a subsequent confirmatory experiment, we examined similarly treated monolayers by confocal microscopy (Fig. 2C to E). As expected, BAF treatment increased the intensity of autophagosomes within a cell while decreasing the intensity of VZV glycoprotein expression within the cell. We have previously shown that levels of VZV gE expression increase for the first 72 h after infection in the absence of BAF (19). Because we saw less viral glycoprotein expression in the presence of BAF, we also probed for the Golgi apparatus in another experiment. When we examined the Golgi apparatus with a specific antibody to GM130, the Golgi cisternae in BAF-treated infected cells appeared to be more disassembled than the Golgi cisternae in untreated infected cells (Fig. 2F and G). However, when we constructed a three-dimensional (3D) image of the Golgi apparatus from a z-stack with Imaris software, the Golgi cisternae were disorganized in both treated and untreated infected cells (Fig. 2F and G). We also produced images of the Golgi apparatus in uninfected cells; the Golgi cisternae labeled with the anti-GM130 antibody were more linear in these nonstressed cells (see Fig. S1 in the supplemental material).

### Titers of VZV in cultures treated with bafilomycin.

**FIG 3:**
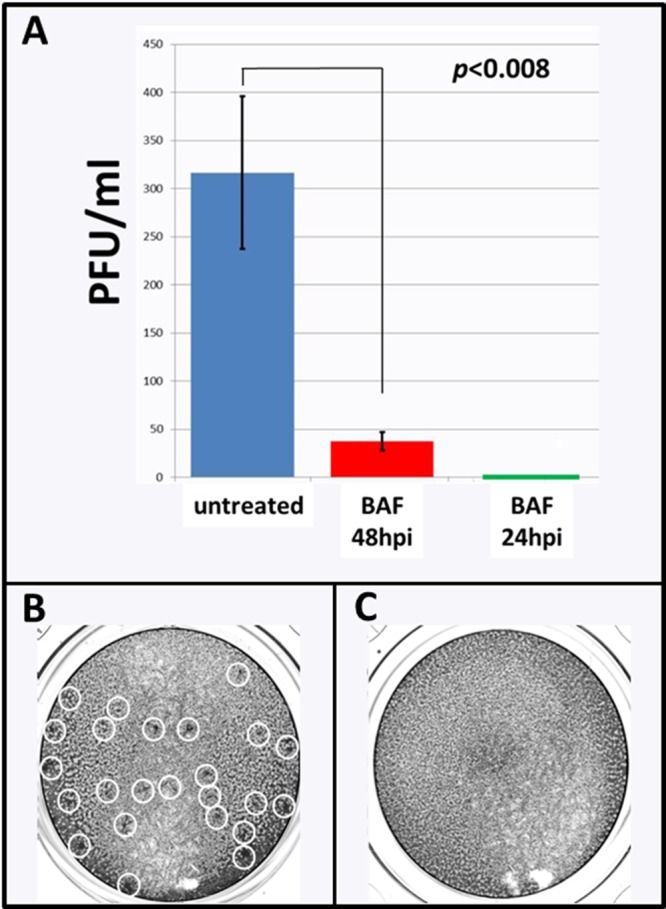
Effect of bafilomycin on titer of VZV cell-free virus. (A) Plaque assays showing titers of VZV in monolayers treated with BAF compared to untreated monolayers. The experiment was repeated 3 times. BAF was added at either 48 hpi or 24 hpi, and the cultures were titrated at 72 hpi. Statistical significance was tested by paired *t* tests and *P* values. Since no plaques were observed when BAF was added at 24 hpi, there is a green bar immediately above the zero bar. (B) One representative well (1:10 dilution) from the plaque assay to titrate VZV from untreated cultures. Plaques are outlined by white circles. (C) One representative well (1:10 dilution) from the plaque assay to titrate VZV from a monolayer treated with BAF for 48 h; no plaques were seen.

Based on the appearance of the monolayer after BAF treatment, along with the decreased gE production, we predicted that the VZV titer in treated infected cells would be lower than the titer in untreated infected cells. To carry out the titrations, we sonically disrupted the cells in the infected monolayer after a 3-day incubation to obtain cell-free VZV. Following this procedure, the infectivity of the sonicate was titrated by 10-fold dilutions (Fig. 3). There was a marked drop in titer in the infected monolayer treated with BAF for 24 h, in other words, from 48 to 72 hpi. The differences in titers between a treated and an untreated monolayer were significant. When the monolayer incubated with BAF for 48 h (24 to 72 hpi) was sonicated and titrated, no infectious cell-free virus was recovered (Fig. 3). Because the decrease in infectivity after BAF treatment was substantial while the confocal microscopy experiments shown in Fig. 2 failed to reveal an explanation, we decided to examine the BAF-treated infected cells by electron microscopy.

### Locations of VZV particles within nuclear membranes and cytoplasm after bafilomycin treatment.

**FIG 4:**
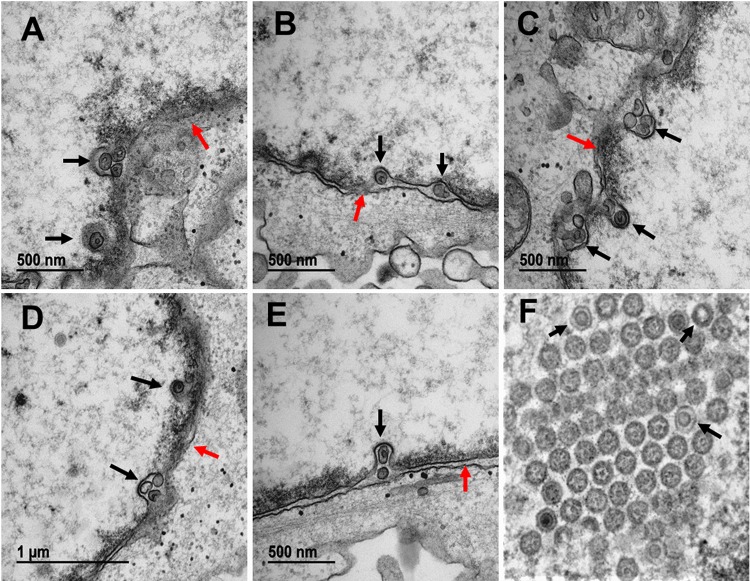
Images of nuclear membranes in BAF-treated VZV-infected cells. BAF (10 nM) was added to the medium for the final 24 h of a 72-h infectious cycle. (A to E) Nuclear membranes in BAF-treated infected cells. Each nuclear membrane contained viral particles in the perinuclear space. The black arrows indicate the locations of capsids. Many capsids had an aberrant appearance. The red arrows indicate the ONM. (F) Nucleus of an infected cell without BAF treatment to show the range of aberrant VZV capsids under conditions of infection without BAF. The most aberrant capsids are indicated with arrows. Aberrant VZV capsids were described in detail previously (20).

**FIG 5:**
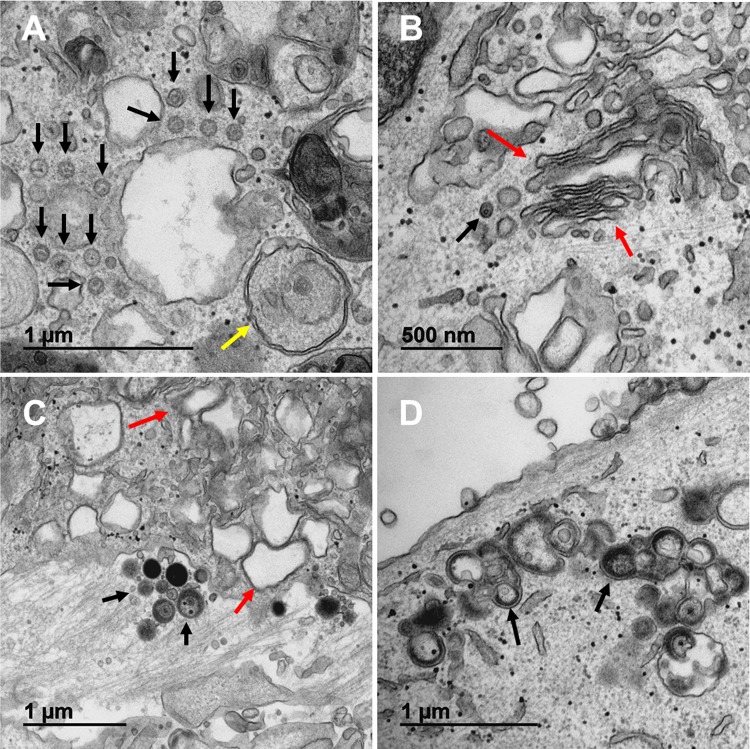
Images of Golgi apparatus and virus assembly compartments in BAF-treated VZV-infected cells. (A) Viral capsids surrounding a Golgi apparatus vacuole. Several capsids surrounding the vacuole are indicated with black arrows. A typical double-walled autophagosome was seen adjacent to the Golgi-derived vacuole (yellow arrow). (B) A Golgi apparatus that is relatively intact in the presence of BAF. The Golgi cisternae are designated with red arrows. One viral particle was seen (black arrow). Note the absence of wrapping of any viral particle within the *trans*-Golgi apparatus. (C) Viral assembly compartment. The VAC contained light particles with varying diameters (black arrows). There were other Golgi-derived vacuoles adjacent to the VAC (red arrows). (D) Virus assembly compartment. The VAC contained approximately 10 light particles with varying diameters. Two of the particles are indicated by arrows.

Based on the abrupt drop in titer in monolayers treated with BAF, we postulated that the defect was occurring during or after secondary envelopment of the viral particle. For this project, we acquired 230 additional micrographs of both BAF-treated infected and uninfected monolayers. The initial observations of BAF-treated infected monolayers provided an unanticipated finding, namely, numerous viral particles were detected in the perinuclear space (Fig. 4). These particles often did not have the typical structure of a primary enveloped virus (PEV); instead, they had an aberrant appearance that had been observed previously by ourselves and others in VZV-infected cells without BAF treatment (20–22). After further inspection, it was apparent that VZV particles were also found in the cytoplasm, often adjacent to Golgi apparatus-derived vesicles (Fig. 5). Some of these capsids also were aberrant in appearance (Fig. 5A). Although most Golgi cisternae were disrupted, an occasional Golgi apparatus with *cis* and *trans* cisternae was found; however, we were unable to find any evidence of wrapping of capsids by intact Golgi cisternae (Fig. 5B). Only after considerable inspection of the 230 micrographs were we able to find a few examples of virus assembly compartments (VACs). The VACs in BAF-treated cells did not contain prototypical enveloped viral particles (virions); instead, they contained enveloped particles without capsids, presumably light particles with varying diameters. The VACs were located near other Golgi-derived vacuoles (Fig. 5C). A particularly large VAC is shown in Fig. 5D; this elongated structure was likely a single VAC that extended below and above the plane cut by the microtome.

As described in Materials and Methods, we have archives of 932 transmission electron micrographs of VZV-infected monolayers without BAF treatment. Each of these transmission electron micrographs was viewed again as part of this investigation (see Fig. S2 in the supplemental material). An occasional transmission electron micrograph included an unenveloped viral particle in the cytoplasm, but we were unable to find clusters of capsids in the cytoplasm. It was uncommon to find more than a single naked viral particle along the outer nuclear membrane (ONM) of most infected cells. An occasional capsid was observed entering the inner nuclear membrane (INM). As a general comment, therefore, there appeared to be a greater number of viral particles in the perinuclear space within BAF-treated monolayers.

As another control, we reexamined the 932 transmission electron micrographs in our archives and selected 24 that were taken of infected cells at 24 hpi. Because of the low titer of input VZV, newly infected monolayers did not show cytopathic effect until around 36 to 48 hpi. Also because of the low titer of input virus, virus entering a cell could not be easily detected by transmission electron microscopy (TEM) at the plasma membrane or at the nuclear membrane during the first 24 hpi (see Fig. S3 in the supplemental material). Thus, the viral particles within the nuclear membrane shown in Fig. 4 cannot be input virus.

### Observations on bafilomycin-treated uninfected cells.

**FIG 6:**
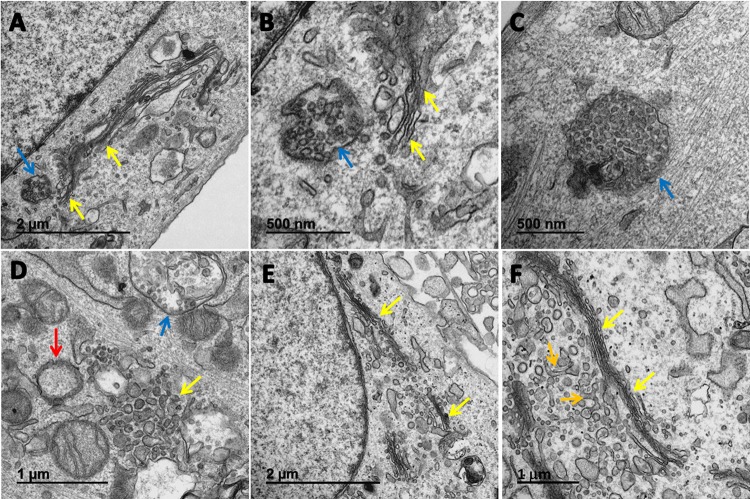
Golgi apparatus and multivesicular bodies in uninfected cells treated with bafilomycin. (A to D) Representative examples of Golgi apparatus and MVBs in BAF-treated cells. Golgi apparatuses are indicated by yellow arrows; MVBs are indicated by blue arrows. (B) Enlargement of panel A to illustrate the MVB near a Golgi apparatus. (C) A single large MVB. (D) A disorganized *trans*-Golgi network with numerous adjacent vesicles and an MVB. There is also an autophagosome near the Golgi apparatus (red arrow). (E and F) Representative examples of Golgi apparatus in untreated cells as a control. (E) A typical ribbon-like Golgi apparatus stack with adjacent vesicles but no MVBs. (F) A typical Golgi apparatus stack. There are tubular TGNs on the *trans* face (orange arrows).

In an extensive search of the literature, we found very few transmission electron micrographs of BAF-treated monolayers that were not further subjected to infection or other stress factors. Therefore, we performed a detailed comparative analysis of BAF-treated uninfected MRC5 monolayers (Fig. 6). BAF treatment led to disorganization and shortening of the Golgi cisternae, often with no discernible *cis* or *trans* face (Fig. 6A to D). Large clusters of vesicles often surrounded the remnants of the cisternae. We observed a markedly increased number of distinctive vacuoles in BAF-treated monolayers, namely, multivesicular bodies (MVBs), which were not readily detectable in the untreated monolayers. MVBs closely resembled in morphology the late endosome/MVB fraction described in an earlier paper using BAF (23). Some MVBs contained over 50 vesicles in a two-dimensional (2D) slice (Fig. 6C). Some investigators have also used the term “amphisome” to describe a larger MVB filled with cargo (24, 25). We also included examples of Golgi apparatuses in uninfected and untreated cells (Fig. 6E and F). Tubular structures typical of the TGN were seen in one micrograph (Fig. 6F). Numerous vesicles were visible near the *trans*-Golgi network, but few MVBs were seen.

### Reexamination of micrographs of BAF-treated infected cells.

**FIG 7:**
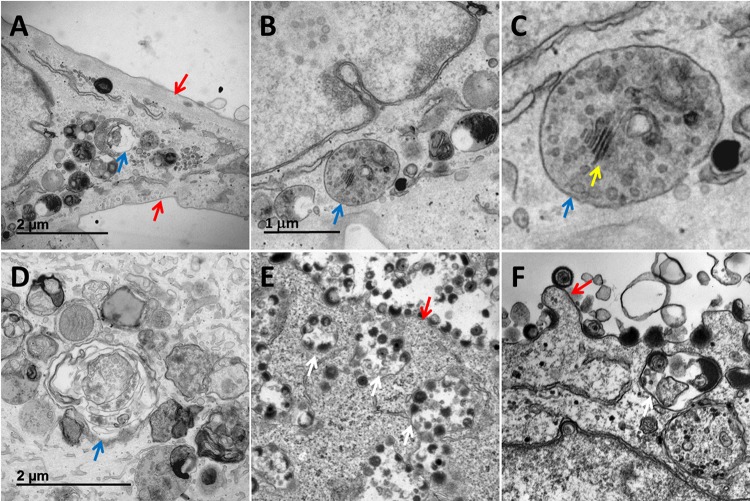
Reexamination of Golgi apparatus in bafilomycin-treated VZV-infected cells. The organelles in the cytoplasm in BAF-treated infected cells (A to D) and untreated infected cells (E and F) were compared. (A) Examples of Golgi apparatus disassembly and MVBs (blue arrow) secondary to BAF treatment. Note the absence of viral particles along the plasma membrane (red arrows). (B) Large vacuole containing both Golgi apparatus vesicles and remnants of Golgi apparatus stacks. (C) Enlargement of vacuole in panel B. Note the easily distinguishable Golgi apparatus remnant stack inside the vacuole (yellow arrow). (D) Numerous vacuoles in the cytoplasm. One vacuole contained Golgi apparatuses. Vacuoles did not contain intact viral particles. (E) Typical appearance of vacuoles containing mainly aberrant VZV particles en route to the plasma membrane in the absence of BAF. Note the abundant viral particles along the plasma membrane (red arrow) and vacuoles with viral particles in the cytoplasm (white arrows). (F) Vacuole with 4 viral particles in the cytoplasm. Both light particles and virions are seen along the plasma membrane. A prototypical VZ virion is located next to a red arrow.

After viewing the BAF-treated uninfected cells, we reexamined the changes in organelles in the cytoplasm of BAF-treated infected cells. In particular, we found disassembly of the Golgi apparatus as a prominent feature (Fig. 7A to D). Intact Golgi apparatuses with stacks of cisternae were rarely present. We were unable to locate any capsids in the cytoplasm that were encircled by Golgi apparatus membranes as evidence of secondary envelopment. We observed numerous MVBs. We also observed an occasional endosome that contained not only vesicles, but also fragments of Golgi apparatus stacks (Fig. 7B and C). Other cytoplasmic vacuoles contained additional, even larger portions of the Golgi apparatus (Fig. 7D). These MVBs were not found in untreated VZV-infected cultures (Fig. 7E and F). We observed a second major difference in BAF-treated infected cultures. By the third day of a typical VZV infection, numerous virus-filled vacuoles were seen in the cytoplasm, and viral particles (both virions and light particles) were seen along the plasma membrane (Fig. 7E and F). In contrast to untreated infected cultures, the cytoplasm in BAF-treated monolayers was not filled with transport vacuoles containing 1 to 4 prototypical enveloped viral particles, and viral particles were not aligned along the plasma membrane (Fig. 7A). These observations further documented that secondarily enveloped viral particles were not seen in BAF-treated cells.

## DISCUSSION

Through the use of the vacuolar proton ATPase inhibitor BAF, this study confirmed the centrality of the Golgi apparatus/TGN as a platform in the VZV infectious cycle. There were two important conclusions, both of which required visualization of the infected monolayers by electron microscopy. The most important conclusion was that the reduced VZV titers secondary to BAF treatment were caused by an apparent failure of secondary envelopment within a VAC. Capsids were transported through the inner nuclear membrane, some of which were detected within the perinuclear space more frequently than was seen in infected cells without BAF treatment. Although many of these particles were aberrant in appearance, a variety of similarly aberrant VZV particles have been seen previously in VZV-infected cultures (20). Capsids exited the ONM and were detected within the cytoplasm, often adjacent to Golgi-derived vacuoles. When we surveyed prior electron microscopy investigations of the VZV infectious cycle, we noted that the virtual absence of VZV capsids in the cytoplasm of untreated infected cells (human amnion cells, human fibroblasts, and human melanoma cells) had been documented by 6 different laboratories over the past 50 years (21, 22, 26–29). Thus, BAF treatment led to an accumulation of capsids in the cytoplasm. We also attempted to find evidence for wrapping of the capsids within the TGN but were unsuccessful after an extensive search. Instead, we found a few Golgi-derived vacuoles containing viral particles that resembled light particles. The particles contained an envelope but no capsid; the particles also varied considerably in diameter. We failed to find prototypical enveloped capsids (virions 200 nm in diameter) within individual vesicles in the cytoplasm. Based on the above-mentioned observations, we propose that secondary envelopment is blocked by BAF treatment. In other words, capsids are transported to the region of the TGN, but the formation of the compartment required for tegumentation and/or envelopment of incoming capsids is disrupted in BAF-treated cells. The above-mentioned VZV data are also supported by models of HSV assembly (30).

**FIG 8:**
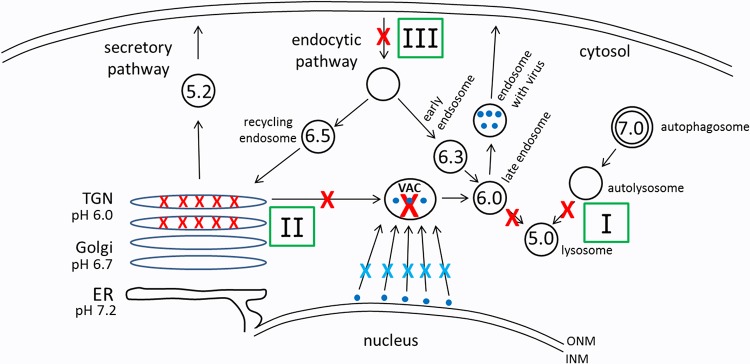
Diagram to illustrate various effects of bafilomycin. BAF causes several alterations in the trafficking pathways due to its inhibition of the vacuolar proton ATPase. Pathway I includes the transition from autophagosome to lysosome. Pathway II includes the TGN. Pathway III includes the endocytosis pathway. The numbers inside the circles indicate pH without BAF. A red X indicates a known site of a BAF-induced block. A blue X indicates a proposed slowing in viral transport between the ONM and the virus assembly compartment. The small blue dots represent viral particles.

The properties of the BAF compound were reported in a 1988 landmark paper (31). BAF is a macrolide antibiotic that inhibits the acid-pumping functions of the vacuolar H^+^ ATPase with high specificity in nanomolar concentrations. Therefore, BAF treatment disrupts the functions of multiple acidic organelles within the central vacuolar system of the cell. Within a decade of the 1988 article, there was a second pivotal paper that reported major impairment of autophagic flux by BAF, namely, BAF treatment prevented fusion between autophagosomes and lysosomes (16) (Fig. 8, pathway I). This effect was presumed to be caused by the inability of the lysosome to maintain an acidic pH in the presence of BAF. The site of attachment of BAF to the ATPase has been determined (31). Subsequently, others have found that this BAF effect involves separate inhibitory events on autophagosome-lysosome fusion and autolysosome acidification (32). Although the autophagic flux studies have received more attention, related BAF studies demonstrated that BAF treatment disrupted the function of the Golgi apparatus, especially the more acidic *trans*-Golgi and TGN (33) (Fig. 8, pathway II). As further proof of its impairment of Golgi apparatus function, several papers reported that BAF treatment interfered with the processing of complex N-linked glycans on enveloped viruses, for example, final processing of the Semliki Forest virus (SFV) and vesicular stomatitis virus (VSV) glycoproteins (34). Additional virology studies showed failure of influenza A and B virus replication in the presence of several different BAF concentrations (35, 36). Likewise, replication of HSV-1 was blocked when it was grown in medium containing 100 nM BAF (37). Taken together, these virology studies clearly showed that one component of the BAF inhibitory effect was impaired envelopment of both RNA and DNA viruses. Similarly, we speculate that BAF treatment of VZV-infected cells drastically reduced the number of virus assembly compartments because of its effects on the TGN (22).

The second conclusion of our BAF studies involves the increased number of MVBs in BAF-treated cells. Investigators in an early study of the origins of MVBs in an animal model (which did not use BAF) concluded that the vesicles inside MVBs can be derived from vesicles found along the convex surface of the *trans*-Golgi cisternae (38). A second group reported that drug-induced disorganization of the Golgi apparatus leads to the release of vesicles mainly from the *trans*-Golgi apparatus (39). A third group documented an increased number of MVBs in cells treated with BAF (40, 41). Specifically, they state that BAF treatment led to a noticeable decline in lysosomes and an increase in multivesicular endosomes. In our transmission electron micrographs, the morphology of the MVBs closely resembled those described by Friend (38) and Mousavi et al. (40). In short, when we examined BAF-treated uninfected and infected cells, we confirmed the prior observation that BAF treatment by itself led to an increased presence of MVBs. We did not include this pathway in Fig. 8, but it would involve entry of Golgi apparatus-derived vesicles released by a disorganized Golgi apparatus secondary to BAF treatment into an endosome by the endosomal sorting complexes required for transport (ESCRT) to form an MVB. Because of the BAF treatment, subsequent fusion of the MVB with a lysosome is blocked.

There are complementary studies of the effects of Golgi apparatus inhibition on the infectious cycles of PRV and HSV-1. Brefeldin A inhibits protein transport from the endoplasmic reticulum (ER) to the *cis*-Golgi (42). In the presence of brefeldin A, PRV crossed the INM and acquired an envelope, becoming a PEV (43), but the passage of the PEVs through the ONM was blocked by brefeldin A. Monensin causes disassembly and vacuolization of the Golgi apparatus (44). When HSV-1 was grown in the presence of monensin, viral titers were decreased and exocytosis of secondarily enveloped particles was limited (45). Thus, the BAF block falls between the locations of the brefeldin block and the monensin block in alphaherpesvirus infectious cycles. We have not yet studied the effect of nocodazole on VZV assembly because the drug also blocks viral entry; therefore, addition of nocodazole to cultures prevents completion of the multistep growth curve over 72 h that is required to achieve a sufficiently high VZV titer to allow visualization of viral particles by TEM (46).

In addition to the newly recognized effects described in this report, BAF has been an important drug in investigations of herpesvirus entry (Table 1). In the HSV entry experiments, BAF was added shortly after virus inoculation (47) (Fig. 8, pathway III). Entry of HSV-1 strain KOS into HeLa cells was inhibited by incubation in the presence of 50 nM BAF at 2 hpi, but BAF treatment did not exert a similar effect on HSV-1 entry into Vero cells. The authors concluded that successful entry of HSV-1 into some (HeLa) cells requires endocytosis and trafficking to an acidic intracellular endosome, while entry into Vero cells proceeds by fusion of the HSV-1 envelope glycoproteins to the plasma membrane. A similar mode of VZV entry by endocytosis rather than fusion has been demonstrated during VZV infection of CHO cells in the presence of 100 nM BAF (48). Finally, our data contradict several findings in a prior report about BAF effects on the VZV infectious cycle; the report did not include any electron microscopy of their BAF-treated VZV-infected cells (49).

## MATERIALS AND METHODS

### Cell culture and virus.

MRC5 fibroblasts (ATCC) and MeWo cells (Sloan Kettering Institute) were grown in minimum essential medium (MEM) (Life Technologies) supplemented with 7% fetal bovine serum, l-glutamine, nonessential amino acids, and penicillin-streptomycin. When the cells were ∼90% confluent, they were inoculated with trypsin-dispersed infected cells at a ratio of one infected cell to eight uninfected cells (18). The titer of the inoculum was 1 million infectious foci/ml. The virus strain was the completely sequenced low-passage-number VZV-32 strain (50).

### Reagents.

Bafilomycin A1 (B1793), as well as rabbit antibody against LC3 (L7543), was purchased from Sigma. The toxicity of BAF to cells was assessed by trypan blue exclusion. The Golgi apparatus GM130 antibody (PA1-077-A555) was obtained from Thermo Scientific. The mouse monoclonal antibody (MAb) to VZV gE (clone 3B3) was produced in our laboratory (51). Secondary antibodies included goat anti-rabbit Alexa Fluor 488 (A11070; Life Technologies) and goat anti-mouse Alexa Fluor 546 (A11018; Life Technologies). The DNA stain was Hoechst 33342 (Invitrogen; H3570).

### Gradient sedimentation and Western blotting.

The protocol for sedimentation of VZV sonicates in potassium tartrate-glycerol gradients has been described previously; the virion bands from the gradients were pelleted, and the supernatant was discarded (52). The pellets were solubilized in SDS-PAGE sample buffer (0.125 M Tris, pH 6.8, 2% SDS, 10% beta-mercaptoethanol, 20% glycerol, and bromophenol blue). The solubilized, purified virus was separated on 4 to 20% gradient SDS-PAGE gels and transferred to polyvinylidene difluoride (PVDF) membranes. The membranes were blocked with phosphate-buffered saline (PBS) with 0.05% Tween 20 containing 5% nonfat dry milk for 30 min at room temperature and then incubated in primary antibodies diluted 1:2,000 in PBS-0.05% Tween 20-1% milk for 1 h at room temperature. The membranes were washed 5 times for 5 min each time at room temperature in PBS-0.05% Tween 20, incubated in goat anti-mouse or goat anti-rabbit horseradish peroxidase (HRP)-conjugated secondary antibodies, diluted 1:25,000 in PBS-0.05% Tween 20-1% milk for 1 h at room temperature, and washed 5 times for 5 min each time at room temperature in PBS-0.05% Tween 20. The labeled proteins were detected using a SuperSignal West Pico Plus chemiluminescent substrate kit from Thermo Scientific.

### Confocal microscopy.

Cells were plated on glass coverslips in 6-well tissue culture plates and incubated at 37°C until they were 90% confluent. The cells were inoculated with VZV-infected cells at a ratio of 1 infected cell to 8 uninfected cells. The infected cells were treated with 10 nM bafilomycin at 24 or 48 hpi. At 72 hpi, the cells were fixed with 2% paraformaldehyde with 0.02% Triton X-100 at room temperature for 1 h. The fixed cells were washed 5 times for 5 min each time in PBS and blocked for 30 min in PBS with 5% nonfat dry milk. The coverslips were incubated in primary antibodies diluted 1:2,000 in PBS-1% milk for 1 h at room temperature and washed 5 times for 5 min each time in PBS. The coverslips were then incubated in goat anti-mouse or goat anti-rabbit secondary antibodies conjugated to Alexa Fluor 488 or Alexa Fluor 546, washed 5 times for 5 min each time, and mounted on glass slides. Images were collected on a Zeiss 710 laser scanning confocal microscope (6). Z-stacks of 2D images were converted into 3D images with Imaris software (Oxford Instruments).

### Transmission electron microscopy.

Cell culture medium overlying a monolayer was replaced with 2.5% glutaraldehyde in 0.1 M cacodylate buffer prewarmed to 37°C (53). The cells undergoing fixation were allowed to cool to room temperature and then remained in the fixative overnight at 4°C. After rinsing several times in 0.1 M cacodylate buffer, postfixation was carried out for 20 min with 1% osmium tetroxide solution reduced with 1.5% potassium ferrocyanide in 0.1 M cacodylate buffer solution (54). This second fixative was removed with several rinses of distilled water. An *en bloc* staining with 2.5% uranyl acetate was done for 5 min. The cells were then dehydrated using gradually increasing concentrations of ethanol to 100%. At this point, infiltration of a 1:1 mixture of Eponate 12 epoxy resin (Ted Pella) and ethanol was applied and incubated for 1 h at room temperature. This mixture was replaced with 100% resin three times at 1-h intervals and cured for an additional 48 h in a 60°C oven. Sections 80 nm thick were cut using a Leica UC-6 ultramicrotome and collected on Formvar-coated 75-mesh copper grids. Counterstaining was done with 5% uranyl acetate for 2 min and with Reynold’s lead citrate for 2 min (55). Images were collected at 120 kV using a JEOL 1230 transmission electron microscope and recorded directly in digital format with a Gatan 2,000-by-2,000 charge-coupled-device camera equipped with Gatan Microscopy Suite software.

### Archives of electron micrographs.

We have retained archives of TEM negative prints in the laboratory. For the TEM results concerning the VZV infectious cycle described in a previous article from our laboratory, the published images were selected from a total of 595 micrographs taken during 12 separate experiments (21). For a subsequent series of TEM analyses carried out as part of the research for a master’s thesis, a total of 337 digital micrographs of VZV-infected cells were retained on file on a hard drive (56).These images were captured during 10 separate experiments. The latter study included our early analyses of autophagy during VZV infection (7). We reexamined all 932 images as part of the current study. In addition, 230 new images were collected as part of the BAF research protocol on infected monolayers.

### Statistical analysis.

The statistical analyses shown in Fig. 3 were performed with Student's *t* test. The test has been described in detail previously (57).

## Supplementary Material

Supplemental file 1
