# Supplementary material for: Progeny Varicella-Zoster Virus Capsids Exit the Nucleus but Never Undergo Secondary Envelopment during Autophagic Flux Inhibition by Bafilomycin A1
Source: J Virol. 2019 Aug 13;93(17):e00505-19. doi: 10.1128/JVI.00505-19 (PMC6694825; doi:10.1128/JVI.00505-19)

Supplemental Figure 1. Immunolabeling of Golgi in uninfected cells. As described, nearly confluent monolayers of uninfected cells were labeled with antibody to the GM130 Golgi protein. The cells were subsequently imaged by confocal microscopy; z-stacks of images were converted into 3D images by Imaris software.

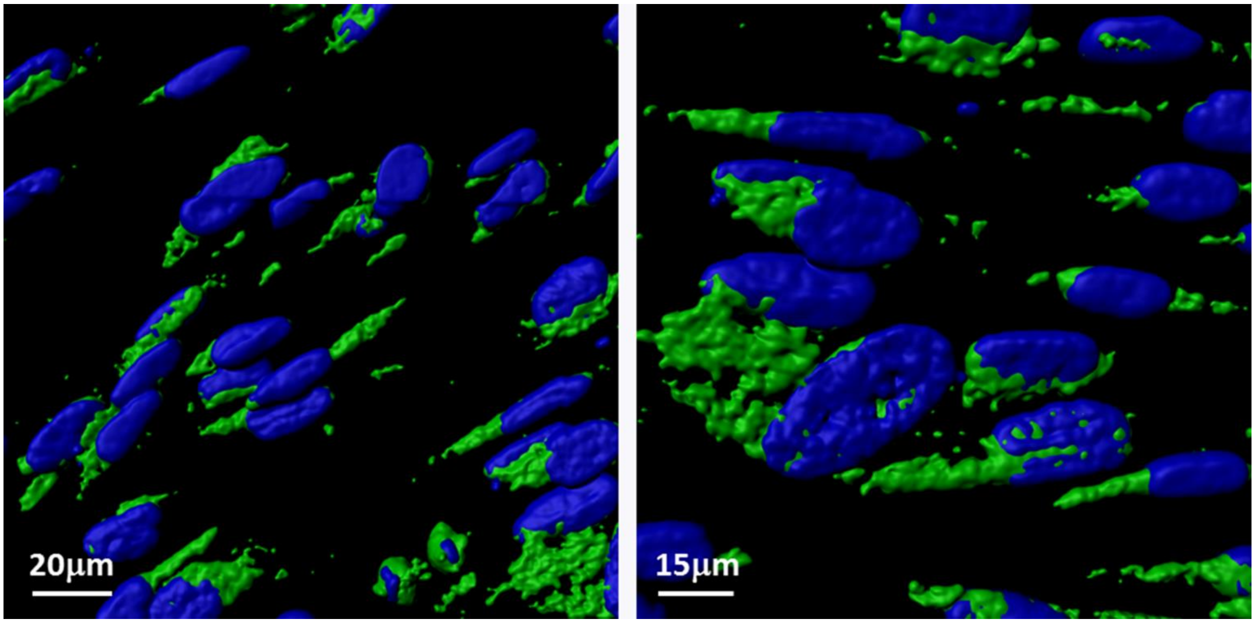

Supplemental Figure 2. VZV infected cells at 72 hpi without bafilomycin treatment. A total of 932 TEM images of untreated VZV-infected monolayers were examined. Six representative images were selected. A-C. Infected cells with no capsids immediately adjacent to the ONM. D,E. Infected cells, each with one capsid adjacent to the ONM (white arrow). F. Infected cell with a capsid passing into the inner nuclear membrane but not yet within the perinuclear space (white arrow). Nuclear membranes are designated with yellow arrows.

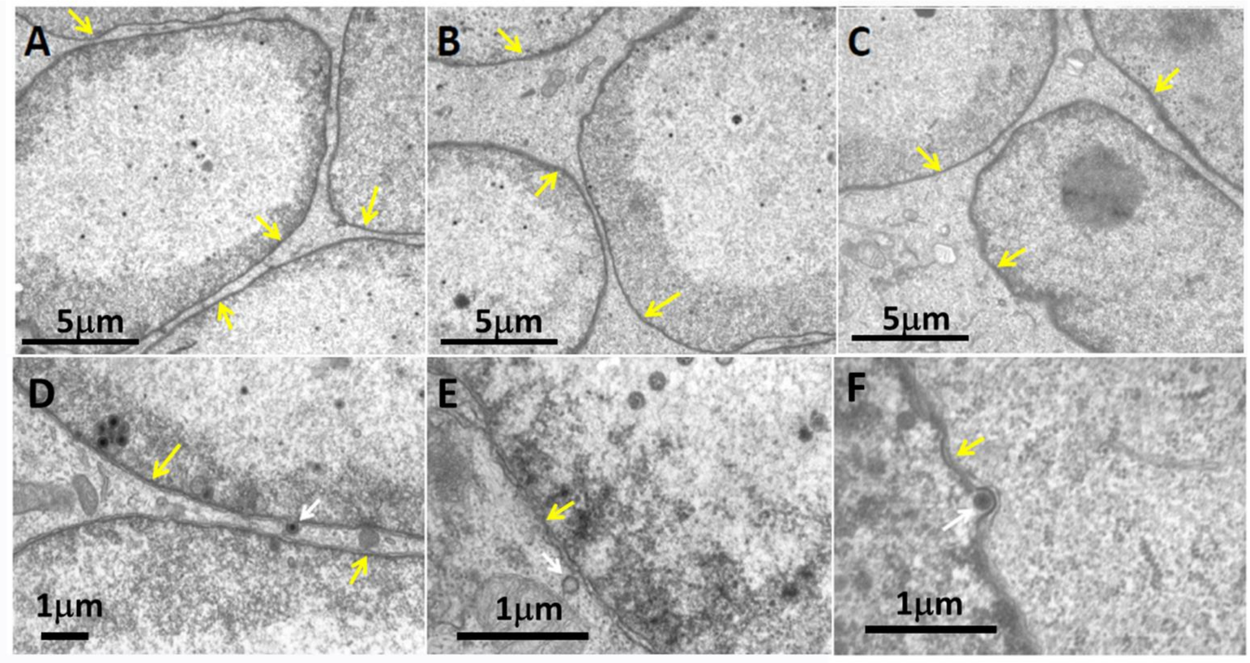

Supplemental Figure 3. Absence of detectable virus by electron microscopy during first 24 h after VZV infection. Because the titer of VZV input virus is invariably low, at least 4 to 5 replication cycles are required before progeny virus is easily detected by TEM. This TEM was taken at 24hpi (replication cycle = 14 to 18 h.). The input virus was not detectable on or beneath the plasma membrane or at the nuclear membrane (yellow arrows); progeny capsids were not detectable within the nucleus. This TEM is a representative example of 24 TEMs taken at 24 hpi and saved in our archives.

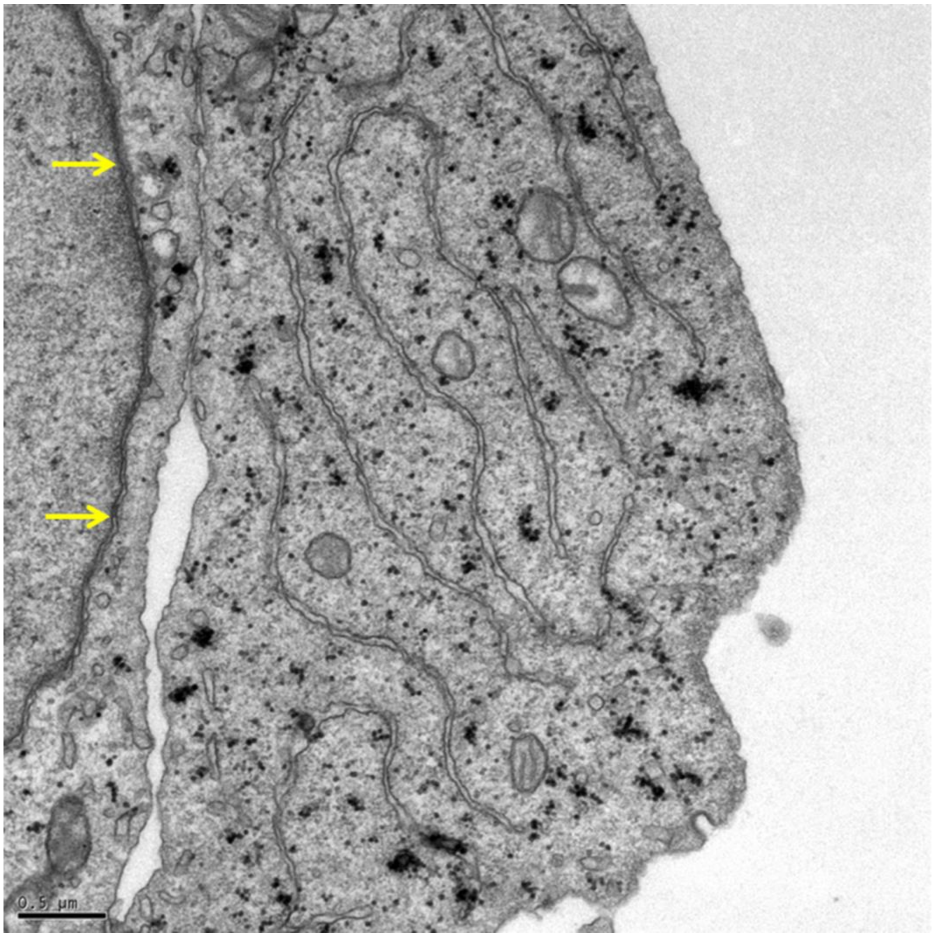

Supplement: Supplemental file 1 [file JVI.00505-19-s0001.pdf]
